# Comparison of Short Uncemented Metaphyseal Stem and Long-Stem Reverse Shoulder Arthroplasty in Proximal Humerus Fractures: Preliminary Study at 2-Year Follow-Up

**DOI:** 10.3390/jcm13164665

**Published:** 2024-08-08

**Authors:** Giorgio Ippolito, Riccardo Maria Lanzetti, Sergio Ferraro, Valerio Pace, Marco Damo, Michele Francesco Surace, Alessio Davide Enrico Giai Via, Michele Crivellaro, Giancarlo De Marinis, Marco Spoliti

**Affiliations:** 1 Trauma & Orthopaedic Department, Icot Hospital “Marco Pasquali”, 04100 Latina, Italy; 2 Orthopaedics and Traumatology Unit, Department of Emergeny and Acceptance, Azienda Ospedialiera San Camillo-Forlanini, 00152 Rome, Italymarcodoc@me.com (M.S.); 3 Asl1 ssr Liguria, 18039 Sanremo, Italy; 4 Trauma & Orthopaedics Department, AOSP Terni, 05100 Terni, Italy; 5 Department of Biotechnology and Sciences for Life, University of Insubria, Cittiglio-Angera, 21100 Varese, Italy; 6 Orthopaedic and Traumatology Department, Orthopaedic and Trauma Center, University of Turin, 10126 Turin, Italy

**Keywords:** reverse shoulder arthroplasty (RSA), proximal humeral fracture, revisions, rotator cuff arthropathy, osteoarthritis

## Abstract

**Introduction**: In the last few years, short metaphyseal-socket prosthetic humeral stems have been introduced for reverse shoulder arthroplasty (RSA). A short stem may have advantages in humeral force distribution, reducing shear stress and preserving bone stock, keeping in mind the need for possible future revision surgery. The main objective of our study was to validate the use of a short stem prosthesis in the surgical treatment of humeral fractures by comparing clinical and radiological outcomes of our studied implant with those obtained with the use of traditional long-stem implants. **Methods**: In this multicentric, controlled prospective study, 125 patients with proximal three- or four-fragment humerus fractures were selected and treated with RSA. A short stem was used in group A (n = 53, mean age: 75.6 ± 5.6 years old), and a long stem was used in group B (n = 72, mean age: 71.76 ± 3). Active range of motion (ROM), Constant score (CS), Quick DASH, American Shoulder and Elbow Surgeons Shoulder (ASES) score, and Visual Analog Scale (VAS) scores were collected and analyzed at 2 years mean follow-up, as well as humeral and glenoid bone resorption (sum Inoue scores and Sirveaux scores were used). **Results**: No statistically significant differences were observed between group A and B in ROM, Constant score (51.69 ± 15.8 vs. 53.46 ± 15.96, *p* > 0.05), Quick DASH (31.5 ± 21.81 vs. 28.79 ± 13.72, *p* = 0.85), ASES (82.53 ± 17.79 vs. 84.34 ± 15.24, *p* = 0.57), or the VAS (0.53 ± 1 vs. 0.56 ± 1.07, *p* = 0.14) at the final follow-up. No statistically significant differences were found in the radiographic parameters between the two groups. No statistically significant differences were found for the average degree of humeral and glenoid bone resorption either. **Conclusions**: The use of a short metaphyseal-socket stem can be considered a safe, effective, and feasible option in reverse shoulder arthroplasty for treating proximal humerus fractures. Our results are encouraging, with no statistically significant differences identified between the proposed treatment and traditional long stems.

## 1. Introduction

Proximal humerus fractures account for 4–10% of fractures, representing a growing problem, especially in the elderly population [1,2]. Among the available treatment options, reverse total shoulder arthroplasty (RSA) is gaining momentum; its utilization rate increased by 191.3% between 2011 and 2017, with an overall incidence rate higher than those of total knee arthroplasty (TKA) and total hip arthroplasty (THA) [3]. Surgical indications for RSA include fractures where synthesis is not possible, especially in elderly patients with poor bone quality and in patients with a proximal humerus fracture and a concomitant massive, irreparable rotator cuff injury [4].

In the last few years, shorter metaphyseal-socket prosthetic stems have been introduced for RSA [5]. A short stem may have advantages in humeral force distribution, reducing shear stress and preserving bone stock, keeping in mind the need for possible future revision surgery [5,6,7,8]. These aspects are increasingly relevant in a world in which life expectancy is constantly increasing and the functional demands of many elderly patients are often higher than in the past.

Despite these potential benefits and the encouraging discussion, there is currently no high-level evidence on the use of short stems in the treatment of proximal humerus fractures available in the literature. The most common practice is to use longer stems, which are supposably able to provide greater primary stability along with their ability to engage the humeral diaphysis [4].

The aim of this study was to demonstrate that short stems have comparable clinical, functional, and radiological outcomes to long stems in the treatment of proximal humerus fractures with RSA.

## 2. Materials and Methods

### 2.1. Study Design and Setting

A non-randomized, multicenter prospective study was conducted, including 125 patients with 3- to 4-fragment proximal humerus fractures enrolled between December 2020 and May 2022. All patients were admitted and treated at two centers: the ICOT Marco Pasquali Hospital in Latina and the San Camillo Forlanini Hospital in Rome, Italy. A CT (computed tomography) scan was performed for all patients, allowing for a better study of the fracture pattern and classification for preoperative planning. All fractures were classified according to the Neer classification [9] and the AO classification [10] by a senior surgeon. Surgery for reverse shoulder arthroplasty was indicated for elderly patients and patients with a high risk of avascular necrosis when the outcome of synthesis was judged unfavorable and when shoulder arthrosis and/or massive cuff tear was present prior to fracture [4]. The exclusion criterion was the presence of comorbidities, which contraindicated performing the surgery. All patients were divided into two independent groups (group A and group B) with similar demographic characteristics and risk factors.

All surgical procedures were performed by a senior surgeon at each institute. All patients enrolled at the ICOT Marco Pasquali Hospital in Latina were treated with short metaphyseal-socket stem RSA (group A). Patients enrolled at the San Camillo Forlanini Hospital in Rome were treated with long stem RSA. In the end, 53 patients were enrolled in group A and the remaining 72 patients were enrolled in group B. All subjects gave their informed consent for inclusion before they participated in the study. This study was conducted in accordance with the Declaration of Helsinki, and its design was approved by the Ethics Committee/Scientific Council of the ICOT Marco Pasquali Hospital in Latina, Italy (protocol no. 1; 18 November 2020).

### 2.2. Surgical Techniques

**Figure 1:**
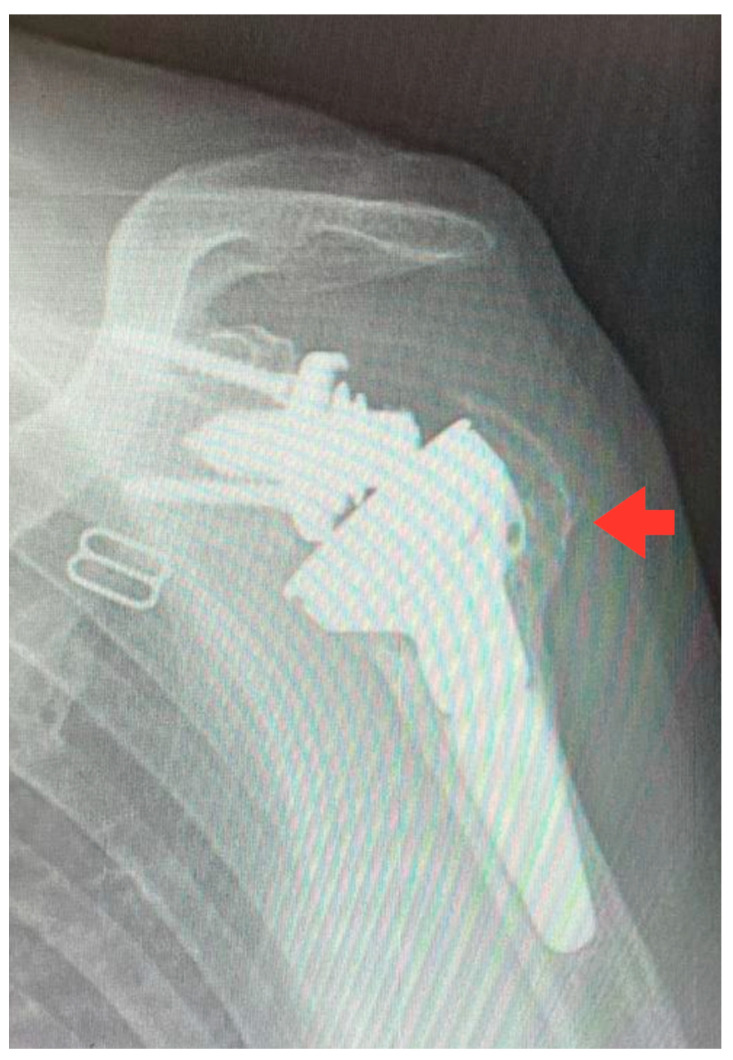
Short-stem RSA at 2-year follow-up. Notice the healing of the great tuberosity (red arrow).

**Figure 2:**
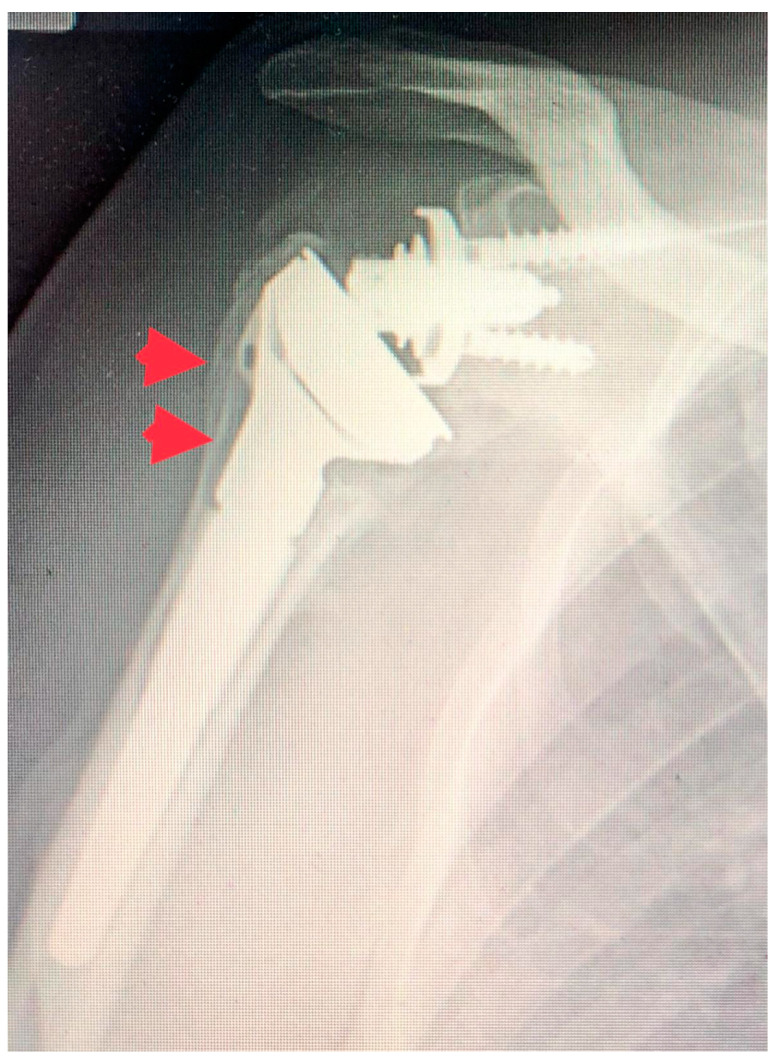
Long-stem RSA at 2-year follow-up. Notice the healing of the great tuberosity (red arrow).

All surgical procedures were performed using a delto-pectoral approach, taking care to avoid neurovascular injuries, deltoid damage, and detachment from the origin. All patients received the same prosthetic system, the SMR uncemented reverse shoulder system by LIMA Corporate^®^. In group A, a short metaphyseal-socket stem was chosen (Figure 1), while in group B, a longer stem with a more diaphyseal socket was utilized (Figure 2). In both implants, the neck–shaft angle was 145°. The short stem length was 45 mm and the long stem length was 80 mm, with the humerus positioned at 20° of retroversion. The glenoid implants consisted of a 40 mm or 44 mm cross-linked ultrahigh-molecular-weight polyethylene (X-UHMWPE) glenosphere, with a central pegged trabecular titanium metal-back, stabilized with two cortical screws. Press-fit fixation was employed to secure the prostheses in all patients. Additionally, the greater tuberosity was repaired in every patient, as well as the lesser tuberosity with the subscapularis tendon using nonabsorbable sutures (size 2).

### 2.3. Clinical, Functional, and Radiological Follow-Up

Regular wound reviews and removal of sutures at 2 weeks post-op were provided for all patients in the clinics. Patients underwent periodical clinical, functional, and radiological evaluation (true anteroposterior and axillary views) at 1, 3, 6, and 12 months, with a final follow-up at 2 years. Patients were evaluated by a senior surgeon and X-rays were reviewed by a radiologist from each center involved in this study.

### 2.4. Post-Operative Rehabilitation Protocol

All patients in both groups followed the same post-operative rehabilitation protocol in the same rehabilitation center. Patients were instructed to wear a shoulder brace with 15° of abduction for 4 weeks. During this time, gentle, gravity-assisted pendulum exercises were initiated starting from post-operative day 15. External rotation was limited until week 4. From week 4 to week 8, patients began passive range of motion (ROM) exercises and gradually progressed to active ROM exercises over the course of 6 weeks, with the aid of a physiotherapist. This period of rehabilitation aimed to restore shoulder mobility and function. After 8 weeks, patients were encouraged to continue independent home exercises to further enhance their recovery. The standardized post-operative protocol ensured consistent rehabilitation practices for all patients in the study.

### 2.5. Outcomes

**Figure 3:**
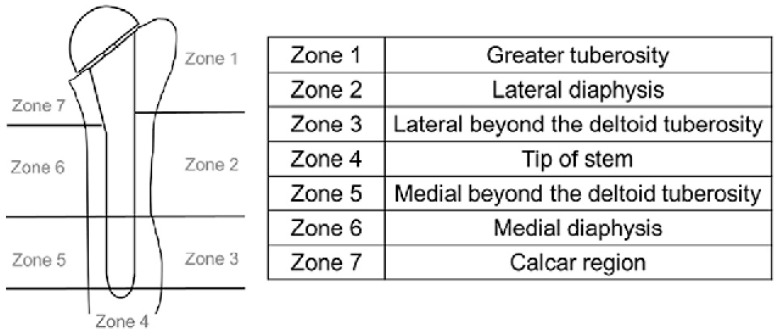
Inoue division of the proximal humerus.

Active ROM was measured in terms of anterior elevation, abduction, external rotation with the arm adducted, and internal rotation with the arm abducted. Clinical scores were used to assess shoulder function and patient-reported outcomes. The scores used in this study included the Constant Score (CS), Quick DASH, American Shoulder and Elbow Surgeons Shoulder (ASES) score, and Visual Analog Scale (VAS) score. Radiographic evaluation was performed by examining true anteroposterior and axillary views. All patients were evaluated at the final 2-year follow-up period. An experienced shoulder arthroplasty surgeon, who was not involved in the procedures, evaluated the radiographic parameters according to the method proposed by Inoue et al. [11] (Figure 3). Notching was assessed according to the methods of Sirveaux et al. [12]. To ensure accuracy in the analysis, radiographs from the last follow-up were compared with those taken immediately after the surgery. This allowed for easier identification of signs of loosening. To avoid errors in measuring the thickness of the radiolucency lines, their size was calibrated to that of the prosthetic stems, which have a known size. When radiolucent lines wider than 2 mm were observed, involving three or more zones of the prosthesis, that was considered an indication of potential risk of implant loosening.

### 2.6. Complications

Complications such as dislocations, adhesive capsulitis, nerve injuries, surgical site infections, and cases requiring revision procedures were carefully recorded and monitored throughout the study.

### 2.7. Statistical Analysis

We estimated the sample size required to detect significant outcome variations (Quick DASH, Constant, ROM, and VAS) using a power analysis prior to the study. We used a 2022 Cochrane systematic review [13] as a reference for mean outcome and clinically important variation values.

We estimated that the sample size required to achieve a power of 0.8 with an effect size of 0.5 and alpha error of 0.05 was from 50 to 63.

The normality of the data was assessed using skewness and kurtosis tests, with the results indicating acceptable normality (skewness < 1 and kurtosis < 3). Additionally, the Shapiro–Wilk test was performed, yielding a *p*-value of approximately 0.15, suggesting that the data were normally distributed.

The data are presented as means and standard deviations for continuous variables. Differences between the groups were analyzed using pooled variance *t*-tests, with a critical t-value set at 0.05, comparing all the outcomes detected.

Statistical analyses were conducted using Jamovi (The Jamovi project (2024). Jamovi (Version 2.3) [Computer Software]. Sydney, Australia. Retrieved from https://www.jamovi.org, (accessed on 18 June 2024).

## 3. Results

### 3.1. Clinical Outcomes

The demographic characteristics and radiological classification are reported in Table 1.

No statistically significant differences were observed in any of the reported outcomes concerning active ROM or for the subjective scores submitted to the patients (Table 2).

No statistically significant differences were found in terms of elevation, abduction, external rotation, and internal rotation (*p* > 0.05).

The Constant score values were 53.46 ± 15.96 in group B and 51.69 ± 15.8 in group A. However, no statistically significant difference was found between the two groups (*p* = 0.61). Similar results were obtained when comparing the Quick DASH, ASES, VAS, and SST scores between the two groups (*p* > 0.05). No clinical signs of stem loosening were found in any patient.

### 3.2. Radiological Outcomes

No statistically significant differences were found in the radiographic parameters between the two groups at the final 2-year follow-up (Table 2).

### 3.3. Complications

One patient in each of the two groups was diagnosed and treated for shoulder dislocation in the follow-up period at 2 and 4 months from surgery, respectively, both following a traumatic event. They were both successfully treated with a closed reduction procedure under sedation and a splint after reduction for 4 weeks. Full recovery was achieved in both cases. Periprosthetic fracture, acromion fracture, periprosthetic joint infection, and axillary nerve palsy were not reported in either group. No other complications were recorded.

## 4. Discussion

The main finding of this study is that there are no differences between short metaphyseal-socket prosthetic stems and long stems in terms of outcomes and radiological findings when performing RSA for proximal humerus fractures.

The management of proximal humerus fractures presents a complex challenge for orthopedic surgeons, and the indications for appropriate surgical treatment are constantly evolving. Several factors need to be considered, including patient-specific factors, fracture pattern, and advancements in technology [4,14].

The need for revision surgeries has also become more frequent, due to the increasing prevalence of shoulder replacements and the aging population [3]. This factor necessitates careful consideration during preoperative planning, as the consequences of different surgical choices and implant types must be taken into account [2,3,4,5,15,16,17].

Traditional implants used for reverse RSA typically feature a long, cemented stem. This design aims to provide enhanced primary stability of the stem and better control of humeral height, version, and varus valgus orientation [1,2,3,4].

Over the last few years, intense research has been conducted towards the development of new implant types that focus on preserving bone stock and optimizing force distribution, thereby eliminating the need for cement in certain specific cases.

These implants offer several advantages compared to traditional cemented implants, including reducing stress shielding, zeroing the stresses at the diaphyseal level, decreasing the likelihood of periprosthetic fractures with complex patterns, and facilitating easier implant removal during revision surgeries [5,6,7,8].

### 4.1. Clincal Outcomes

In our study, the results regarding the recovery of active ROMs in both short-stem and long-stem patients align with the findings reported in the literature. No statistically significant differences were observed for any of the parameters, including elevation, abduction, external rotation, and internal rotation. The recorded values in the short-stem group A were satisfactory, and similar results have been reported in previous studies. For instance, Dukan et al. [18] obtained comparable values in active elevation and external rotation, with 131° and 36°, respectively. Peduzzi et al. [19] and Goetzmann et al. [20] also reported elevation, abduction, and external rotation values similar to ours, with results of 131° elevation, 111° abduction, and 28° external rotation in the study by Peduzzi et al. [19] and 139° elevation, 122° abduction, and 28° external rotation in the study by Goetzmann et al. [20].

Regarding long stems, our average results are consistent with the works of Bühloff et al. [21] and Werner et al. [22], who reported mean elevation values of 120° and 124° ± 31°, and mean abduction values of 110° and 110° ± 29°, respectively. Comparable clinical outcomes were reported in a study by Jonsson et al. [23] conducted about long-stem RSA. The patients in their study had a mean flexion of 125°, abduction of 112°, and external rotation of 18° rotation.

The subjective scores obtained in our study, such as the Visual Analog Scale (VAS), Constant score (CS), and American Shoulder and Elbow Surgeons Shoulder (ASES) score, are also in line with the findings from the literature [18,22,24].

Dukan et al. [18] reported mean values similar to ours, with a mean Constant score of 87.9, a mean ASES score of 84.3, and a mean VAS score of 0.4. Nourissat et al. [24], at a mean follow-up of 6.7 ± 0.5 years, reported values of 34.7 ± 21.2 for CS and 87.9 ± 13.7 for ASES score. Similarly, the data available for long stems in the literature align with the results obtained in our study (the results can be seen in Table 2). For example, Werner et al. [22] reported mean CS values of 39.5 ± 13.7, while the study by Jonsson et al. reported a mean Constant score of 58.7 points [23].

### 4.2. Radiological Outcomes

The radiological results recorded and reported in our study are encouraging, demonstrating low resorption values at a follow-up of 2 years, such that no loosening of the prosthesis was noticed in any case and no revision was needed.

Regarding humeral bone resorption, the areas of greater resorption were zones 1, 2, and 7, meaning the tuberosities, the lateral part below the tuberosities, and the calcar, respectively.

The mean value of resorption grade in these areas was less than 1, meaning that only a mild decrease in bone density was recorded. In addition, grade 2 and 3 resorption was found in a few patients and no cases of grade 4 resorption were recorded in either group.

Consequently, relevant grades of resorption were very infrequent and almost never associated with the calcar zone. A slightly higher probability of resorption was noted in the tuberosity zone. These results could be explained by the fact that these areas with higher risk of resorption are where the stresses and shear stress are greatest or the blood supply is poorer.

Comparing our results with those in the literature is challenging, as different radiographic classifications are often used or data are reported purely qualitatively. Several biases and significant heterogeneity also exist.

Aibinder et al. [25], for example, classified the stress shielding as mild, moderate, or severe in their study about short-stem elective RSA, reporting 12 (18.46%) cases of stress shielding. Calcar resorption was noted in 16 (25%) shoulders.

In their systematic review, Tross et al. [26] used the Schnetzke et al. [27] definition of bone remodeling, considering condensation lines, cortical bone resorption or osteopenia, and spot welds. They found humeral cortical thinning in 15 of 205 cases reported by four studies. Humeral condensation lines were found in 8 of 205 cases reported in two studies. Humeral spot welds were described by two studies in a stem zone distribution model.

Bone adaptions were categorized according to the area of presentation as “high” and “low” by Schnetzke et al. [27] and Raiss et al. [28], who found high humeral bone adaptions in 15 of 96 cases (10.5% to 17%) and low bone adaptions in 81 of 96 cases (83% to 89.6%), assessing most of these findings in the “under the stem” area. With regard to their severity, Raiss et al. [28] categorized radiographic changes as “severe” (5%), “moderate” (12%), “mild” (18%), and “no changes” (65%).

In the same systematic review, Tross et al. [26] reported that osteolysis and radiolucent lines were present in 25 of 345 cases, as shown in three studies.

In two studies based on the use of long-stem RSA, Jonsson et al. [23] found radiolucent lines wider than 2 mm in three zones in only 1 of 36 patients [24], and Buhlof et al. [21] reported stem loosening in 13.6% of cases.

Regarding greater tuberosity resorption, Merolla et al. [29] reported resorption of the greater tuberosity and lesser tuberosity in 5% of cases in short-stem RSA. In their systematic review, with about 268 patients treated with long-stem RSA instead, Ferrel et al. [30] demonstrated a tuberosity malunion in 4.9% of cases, a nonunion in 8.2%, and resorption in 4.1%. The greater tuberosity was united with the shaft in 67% of patients.

We recorded slightly better results compared to the above-mentioned studies, without any statistically significant difference among the two groups. This aspect supports the use a short-stem RSA, which seems to be a valuable surgical option to treat this cohort of surgical patients.

Ferrel et al. [30] and Bülhoff et al. [21] found scapular notching in 25.4% and 68.2% of patients, respectively, undergoing RSA with a long stem. In the latter, glenoid loosening was observed in two (9.1%) patients and polyethylene wear was observed in four (18.2%) cases.

In the 41 patients who underwent long-stem RSA in the study by Jonsson et al. [23], scapular notching was assessed according to Sirveaux as none (n 28), grade 1 (n 6), or grade 2 (n 2).

Among the short-stem RSA considered in Tross et al.’s [26] study, scapular notching was reported in 18% of patients (60 out of 327), being the most common radiological finding. Glenoid loosening was found in 3 of 352 cases in two studies, with 1 case undergoing revision to exchange the polyethylene insert.

In another recent study about short-stem RSA, Ascione et al. reported a 37% rate of scapular notching, not associated with any adverse clinical outcomes [31].

### 4.3. Complications

Only two significant complications were recorded in our study (one in each group, both successfully treated without any further complications). No infections were recorded in our study.

Comparing our findings to a systematic review by Tross et al. [26], we observed a lower complication rate. This systematic review reported an average complication rate of 12.9% at 32 months FU, indicating that our study had a lower incidence of complications.

Our complication rate was similar to that in the study of Jonsson et al. [23], who reported only 1 case of CPRS in 41 patients at 2 years FU.

In addition, the revision rate of our study was lower than most of the studies already available in the literature that nevertheless have a longer follow-up. Nourissat et al. [24] reported a revision rate of 8%, with two patients requiring revision due to infection and three patients requiring revision due to loosening and glenoid component removal at 6.7 ± 0.5 years FU. Similarly, Bühloff et al. [21] reported a revision rate of 11.1% at 10 years FU. However, it is important to note that those studies refer to elective RTSA procedures, the results of which are different compared to those related to RTSA procedures for humeral fractures. One of the factors that influence this is thought to be the tuberosity healing process. In fact, this process can change the outcomes for the patients. This should be considered by the orthopedic surgeons, who needs to undertake the appropriate measures to optimize this process [32,33].

Another positive aspect to be highlighted is the fact that functional scores and ROM have no correlation with bone resorption and were overall very satisfactory. These results are in line with the results reported in the literature for elective RSA procedures [21,23,30].

Ultimately, it can be stated that there are no substantial differences in radiological findings between long and short stems at short- and medium-term follow-up and that component loosening should not be a concern when using short-stem implants, although new studies remain necessary to reach adequate evidence on their use in fractures [8,26].

To our knowledge, our study represents the first to directly compare short stems with long stems for reverse total shoulder arthroplasty in proximal humeral fractures. We individually compared the clinical, functional, and radiological results obtained with both short-stem and long-stem prostheses, and we also made a comparison with already available results in the related literature.

### 4.4. Limitations

This study has several limitations that should be acknowledged. Firstly, it is a prospective non-randomized study, and randomization of patients could have enhanced the strength of the results. Secondly, the follow-up period is relatively short, being only 2 years. Although the follow-up duration is short, it is important to note that the initial bone integration required for secondary stability typically occurs within the first few post-operative months [34]. Therefore, whilst our study demonstrates the feasibility of using the uncemented short stem in fractures, it does not provide long-term outcome data. Thirdly, it is important to mention that the RTSA procedures were not all performed by the same senior surgeon, which introduces potential variability in surgical technique and expertise.

## 5. Conclusions

The use of a short metaphyseal-socket stem can be considered a safe, effective, and feasible option in reverse shoulder arthroplasty for treating proximal humerus fractures. Our results are encouraging, with no statistically significant differences in comparison to traditional long stems. Furthermore, our results are in line with those available in the literature.

## Figures and Tables

**Table 1:** Demographic characteristics and radiographic classification.

Variable	Group A—Short Stem (*n* = 53)	Group B—Long Stem (*n* = 72)
Age in years ± SD	75.6 ± 5.6	71.76 ± 3
Sex, % (*n*) women	77.35 (41)	69.44 (50)
Diabetes	28.3 (15)	55.6 (40)
Hypertension	67.92 (36)	58.3 (42)
Smoking	30.19 (16)	27.78 (20)
Neer classification of fracture, % (*n*)		
Three-part: Type 8	28.3 (15)	25 (18)
Three-part: Type 9	20.75 (11)	15.28 (11)
Four-part	50.94 (27)	59.72 (43)
AO classification of fracture, % (*n*)		
11B1	22.6 (12)	25 (18)
11B2	18.87 (10)	16.7 (12)
11C1	22.6 (12)	22.2 (16)
11C2	35.85 (19)	36.1 (26)
Surgical time in hours ± SD	1.5 ± 0.4	1.5 ± 0.3

SD: standard deviation.

**Table 2:** Clinical and radiographical results.

Variable	Group A—Short Stem (*n* = 53)	Group B—Long Stem (*n* = 72)	*p*-Value
Elevation in degrees	136 ± 29	131 ± 35	0.75
Abduction in degrees	128 ± 27	126 ± 31	0.36
External rotation in degrees	32 ± 15	32 ± 11	0.17
Internal rotation in degrees	50 ± 21	50 ± 9	0.17
Constant score	51.69 ± 15.8	53.46 ± 15.96	0.61
Quick DASH (%)	31.5 ± 21.81	28.79 ± 13.72	0.85
VAS	0.53 ± 1	0.56 ± 1.07	0.14
ASES	82.53 ± 17.79	84.34 ± 15.24	0.57
Sum Inoue score	2.57 ± 2.99	2.43 ± 2.79	0.33
Zone 1, mean ± SD	0.91 ± 1.26	0.81 ± 1.35	0.38
Zone 2	0.64 ± 0.92	0.61 ± 1.03	0.17
Zone 3	0.06 ± 0.23	0.08 ± 0.40	0.11
Zone 4	0	0	-
Zone 5	0	0	-
Zone 6	0.15 ± 0.36	0.17 ± 0.53	0.24
Zone 7	0.81 ± 0.81	0.74 ± 1.02	0.41
Sirveaux	0.264 ± 0.52	0.278 ± 0.51	0.85

ROM, clinical, and radiological outcomes. American Shoulder and Elbow Surgeons Shoulder (ASES) score and Visual Analog Scale (VAS) score; SD: standard deviation; Disability of the Arm, Shoulder and Hand (DASH).

## Data Availability

The original contributions presented in the study are included in the article, further inquiries can be directed to the corresponding author.
